# Intron Retention and TE Exonization Events in *ZRANB2*

**DOI:** 10.1155/2012/170208

**Published:** 2012-06-17

**Authors:** Sang-Je Park, Jae-Won Huh, Young-Hyun Kim, Heui-Soo Kim, Kyu-Tae Chang

**Affiliations:** ^1^National Primate Research Center, Korea Research Institute of Bioscience and Biotechnology, Ochang, Chungbuk 363-883, Republic of Korea; ^2^Department of Biological Sciences, College of Natural Sciences, Pusan National University, Busan 609-735, Republic of Korea; ^3^National Primate Research Center, Korea Research Institute of Bioscience and Biotechnology, University of Science & Technology, Ochang, Chungbuk 363-883, Republic of Korea

## Abstract

The Zinc finger, RAN-binding domain-containing protein 2 (*ZRANB2*), contains arginine/serine-rich (RS) domains that mediate its function in the regulation of alternative splicing. The *ZRANB2* gene contains 2 LINE elements (L3b, Plat_L3) between the 9th and 10th exons. We identified the exonization event of a LINE element (Plat_L3). Using genomic PCR, RT-PCR amplification, and sequencing of primate DNA and RNA samples, we analyzed the evolutionary features of *ZRANB2* transcripts. The results indicated that 2 of the LINE elements were integrated in human and all of the tested primate samples (hominoids: 3 species; Old World monkey: 8 species; New World monkey: 6 species; prosimian: 1 species). Human, rhesus monkey, crab-eating monkey, African-green monkey, and marmoset harbor the exon derived from LINE element (Plat_L3). RT-PCR amplification revealed the long transcripts and their differential expression patterns. Intriguingly, these long transcripts were abundantly expressed in Old World monkey lineages (rhesus, crab-eating, and African-green monkeys) and were expressed via intron retention (IR). Thus, the *ZRANB2* gene produces 3 transcript variants in which the Cterminus varies by transposable elements (TEs) exonization and IR mechanisms. Therefore, *ZRANB2* is valuable for investigating the evolutionary mechanisms of TE exonization and IR during primate evolution.

## 1. Introduction

Zinc finger, RAN-binding domain-containing protein 2 (*ZRANB2*), also known as ZIS and ZNF265, lies on human chromosome 1p31 and was identified in rat renal juxtaglomerular (JG) cells [1], human [2], and mouse [3]. *ZRANB2* contains 2 zinc finger domains, a C-terminal RS (arginine/serine-rich) domain, a glutamic acid-rich domain, and a nuclear localization sequence [4]. It interacts with components of the splicing factors *U170K*, *U2AF35*, and *XE7* and regulates splicing of *GluR-B*, *SMN2*, and *Tra2*β** [5–7]. The 2 zinc finger domains recognize single-stranded RNA (ssRNA) and bind to a consensus AGGUAA motif [8]. *ZRANB2,* thus, mediates alternative splicing of pre-mRNA, is ubiquitously expressed in various tissues, and is highly conserved from nematodes to humans [3, 5, 9].

Alternative splicing (AS) of premessenger RNAs (pre-mRNAs) is an important molecular mechanism that increases human transcriptome complexity and flexibility [10]. Genome-wide analyses of AS events suggest that 40–60% of human genes have alternatively spliced transcripts [11]. With the aid of accumulated transcriptome sequencing data, 5 distinct AS mechanisms have been identified, including exon skipping, alternative 5′ splice sites, alternative 3′ splice sites, intron retention, and mutual exclusion. Intron retention (IR) events are very rare, accounting for less than 3% of all AS events in the human and mouse genomes [10]. In humans, 14.8% of the 21,106 known genes showed at least 1 IR event, mostly involving untranslated regions (UTRs). Eighty-eight cases of IR events seem to be involved in several syndrome-associated genes and tumorigenic processes [12]. Although exonization is not categorized as 1 of the 5 distinct mechanisms, it represents an AS process by which new exons are acquired from intronic DNA sequences [13]. The generation of canonical splicing sites (splicing acceptor and donor sites) by genomic insertions/deletions or mutations could cause the exonization events [13]. Recent studies have indicated that exonization events are derived from transposable elements (TEs) such as LTR retrotransposons (e.g., human endogenous retroviruses (HERVs)) and non-LTR retrotransposons (e.g., short interspersed elements [SINEs] and long interspersed elements (LINEs)) in various species [14, 15]. Many TE sequences contain potential splice sites [16]. TEs are a major component, comprising more than 40% of the human genome [17]. LINEs and LTR retrotransposons insert themselves into new genomic positions through a copy and paste mechanism by encoding their own reverse transcriptase [18]. SINEs do not have their own reverse transcriptase domain; however, they are reverse transcribed and inserted into the genome by LINE element-enzymatic machinery [13]. TEs regulate gene activity by providing promoter, enhancer, exonization, and new polyadenylation signals [19]. Thus, various genes are regulated by TEs [20]. Nekrutenko and Li suggest that TEs are located in ~4% of protein-coding regions in the human genome [21]. According to the genome-wide survey by Sela et al., 1824 human genes have TE-derived exons; SINE and LINE elements comprise approximately 68% and 18% of exonized TEs, respectively, [22]. Although LINE elements are present in fewer copies and mediate exonizations at a lower frequency than SINE elements do, exonization events of LINEs frequently occur in the human genome. LINEs comprise up to 20% of the human genome [23]. LINE1, LINE2, and LINE3/CR1 are 3 distantly related LINE families that represent approximately 17%, 3%, and 0.3% of the human genome, respectively, [23]. Thus, transposable elements and intronic sequences may serve as transcript units to enrich the transcriptome with limited genomic resources [13].

We performed evolutionary and comparative analyses of rhesus, crab-eating, and African-green monkeys and marmosets to investigate exonization events derived from insertion of LINEs and IR in the protein-coding regions of human *ZRANB2*.

## 2. Materials and Methods

### 2.1. RNA and Genomic DNA Samples 

Total RNA from human (*Homo sapiens* cerebrum, colon, liver, lung, kidney, and stomach) and rhesus monkey (*Macaca mulatta* cerebrum, colon, liver, lung, kidney, pancreas, and stomach) were purchased from Clontech. Total RNA from crab-eating monkey (*Macaca fascicularis* cerebrum, colon, liver, lung, kidney, pancreas, and stomach), African-green monkey (*Cercopithecus aethiops* cerebrum, colon, liver, lung, kidney, pancreas, and stomach), and marmoset (*Callithrix jacchus* cerebrum, colon, liver, lung, Kidney, and stomach) were extracted with the RNeasy mini-kit (Qiagen) and the RNase-Free DNase set (Qiagen). Total RNA samples were provided by the National Primate Research Center (NPRC) of Korea.

We used a standard protocol to isolate genomic DNA form heparinized blood samples from the following species: (1) HU: human (*Homo sapiens*); (2) hominoids, CH: chimpanzee (*Pan troglodytes*), BO: bonobo (*Pan paniscus*); (3) Old World monkeys, RH: rhesus monkey (*Macaca mulatta*), JA: Japanese monkey (*Macaca fuscata*), CR: crab-eating monkey (*Macaca fascicularis*), PI: pig-tail monkey (*Macaca nemestrina*), AF: African-green monkey (*Cercopithecus aethiops*), MA: mandrill (*Mandrillus sphinx*), CO: colobus (*Procolobus sp.*), LA: langur (*Trachypithecus sp.*); (4) New World monkeys, MAR: marmoset (*Callithrix jacchus*), TA: tamarin (*Saguinus midas*), CA: capuchin (*Cebus apella*), SQ: squirrel monkey (*Saimiri sciureus*), NI: night monkey (*Aotus nigriceps*), SP: spider monkey (*Ateles geoffroyi*); (5) prosimian, RL: ring-tailed lemur (*Lemur catta*).

### 2.2. RT-PCR and PCR Amplification 

*ZRANB2* transcripts were analyzed by RT-PCR amplification. M-MLV reverse transcriptase with an annealing temperature of 42°C was used with an RNase inhibitor (Promega). We performed PCR amplification of pure mRNA samples without reverse transcription to demonstrate that the mRNA samples prepared did not contain genomic DNA (data not shown). As a standard control, *RPL32* was amplified by the primers RPL32-S (5′-CAA CAT TGG TTA TGG AAG CAA CA-3′) and RPL32-AS (5′-TGA CGT TGT GGA CCA GGA ACT-3′) from human and rhesus monkey. TE fusion transcript was amplified by the primer pair S1 (5′-GAA ATA TCC CGA CAG GGT TC-3′) and AS1 (5′-GCT GCT TTC TTC AAT GGT CTG-3′) derived from the *ZRANB2* gene (Genbank accession no. NM_005455.4). RT-PCR experiments were carried out for 30 cycles of 94°C for 30 s, 58°C for 30 s, and 72°C for 1 m 30 s. Genomic DNAs from various primates were PCR amplified. The Plat_L3 and L3b elements were amplified by primer pairs S2 (5′-CCC TGT GAC ACG GTG TAG AA-3′) and AS2 (5′-CAG ATC ATT GGG AAT CTG TCC-3′). The 9th exon boundary of *ZRANB2* was amplified by primers S3 (5′-ATA AAA ATC TAA CCC TTG ACT AGG AA-3′) and AS3 (5′-AAA CGT AAA GTC CTG TTA ATG CAG-3′). Genomic PCR experiments were carried out for 30 cycles of 94°C for 30 s, 58°C for 30 s, and 72°C for 30 s.

### 2.3. Molecular Cloning of Genomic PCR and RT-PCR Products and Sequencing Procedure

RT-PCR products were separated on a 1.2% agarose gel, purified with the Gel SV extraction kit (GeneAll), and cloned into the pGEM-T-easy vector (Promega). The cloned DNA was isolated using the Plasmid DNA mini-prep kit (GeneAll). Sequencing of primate DNA samples and alternative transcripts was performed by a commercial sequencing company (Macrogen).

## 3. Results

### 3.1. Structural Analysis of the *ZRANB2* Gene

**Figure 1:**
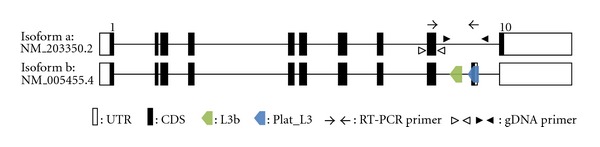
Structural analysis of human *ZRANB2* gene transcripts. The Plat_L3 and L3b elements are located in intron 9. Open and closed boxes represent the untranslated region of the exons and protein-coding region, respectively. Arrows indicate the RT-PCR primer position, and open and closed arrowheads indicate genomic PCR primer positions. These figures are structural illustrations, not to scale.

The *ZRANB2* gene shows two different transcripts (NM_203350.2 and NM_005455.4) in humans according to the GenBank database (Figure 1). Isoform a (NM_203350.2) is composed of 10 exons and is transcribed into a 3070-bp mRNA sequence with a 294-bp 5′ UTR, 993-bp coding sequence, and 1783-bp 3′ UTR. Isoform b (NM_005455.4) is composed of 11 exons and is transcribed into a 3145-bp mRNA with a 294-bp 5′ UTR, 963-bp-coding sequence, and 1888-bp 3′ UTR. The isoform b transcript includes a 196-bp Plat_L3 element belonging to the CR1/LINE3 family from *Ornithorhynchus*. This antisense-oriented Plat_L3 element is inserted between the 9th and 10th exons of *ZRANB2* and provides canonical splicing sites (splicing acceptor and donor site) that produce a new Plat_L3-derived exon via exonization. A premature termination codon (PTC) is generated in the Plat_L3-derived exon of isoform b. The isoform a transcript encodes 330 amino acids, but the isoform b transcript encodes 320 amino acids (Supplemantry Figure  1 in Supplementary Materials available online at doi: 10.1155/2012/170208). These transcript variants possess Ctermini with different amino acid compositions. Another L3b element of the CR1/LINE3 family is integrated in the 5′ upstream intronic region of the Plat_L3 element (Figure 3(f)).

### 3.2. Integration Time of Plat_L3 and L3b Elements

**Figure 2:**
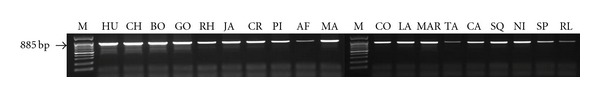
PCR amplification of Plat_L3 and L3b in various primates. Primate DNA samples were utilized for integration analyses of the Plat_L3 and L3b elements in the *ZRANB2* gene. M indicates the molecular size marker. Primate DNA samples are abbreviated as follows: (1) HU: human (*Homo sapiens*); (2) Hominoids: CH: chimpanzee (*Pan troglodytes*), BO: bonobo (*Pan paniscus*), GO: gorilla (*Gorilla gorilla*); (3) Old World monkey: RH: rhesus monkey (*Macaca mulatta*), JA: Japanese monkey (*Macaca fuscata*), CR: crab-eating monkey (*Macaca fascicularis*), PI: pig-tail monkey (*Macaca nemestrina*), AF: African green monkey (*Cercopithecus aethiops*), MA: mandrill (*Mandrillus sphinx*), CO: colobus (*Procolobus sp.*), LA: langur (*Trachypithecus sp.*); (4) New World monkey: MAR: Marmoset (*Callithrix jacchus*), TA: tamarin (*Saguinus midas*), CA: capuchin (*Cebus apella*), SQ: squirrel monkey (*Saimiri sciureus*), NI: night monkey (*Aotus nigriceps*), SP: spider monkey (*Ateles geoffroyi*); (5) prosimian: RL: ring-tailed lemur (*Lemur catta*).

 To determine when the Plat_L3 and L3b elements were integrated during primate radiation, we performed genomic PCR amplification using primer pairs specific to highly conserved regions in various primate samples (Figure 2). We validated the randomly selected amplified products by sequencing (see Supplementary Figure  2). The Plat_L3 and L3b elements in the *ZRANB2* gene were integrated in all tested primate lineages, including hominoids (human, chimpanzee, bonobo, and gorilla), Old World monkeys (rhesus monkey, Japanese monkey, crab-eating monkey, pig-tail monkey, African-green monkey, mandrill, colobus, and langur), New World monkeys (marmoset, tamarin, capuchin, squirrel monkey, night monkey, and spider monkey), and prosimian (ring-tailed lemur).

### 3.3. RT-PCR Amplification and Sequencing Analysis of *ZRANB2* in Human and Monkeys

**Figure 3:**
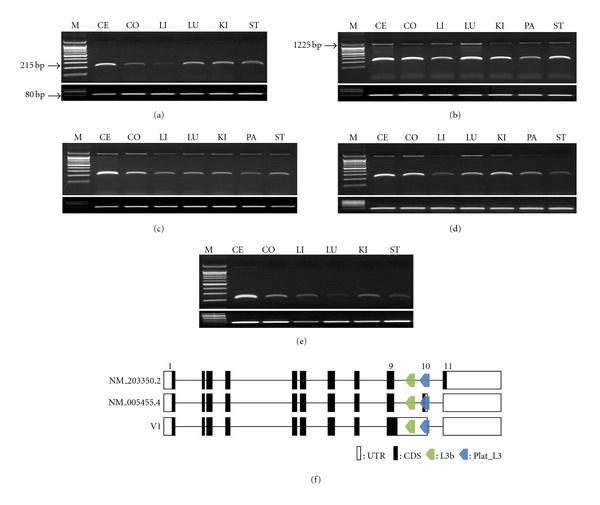
RT-PCR amplification and transcript variants of the *ZRANB2* gene. NM_005455.4 transcripts (215 bp) and V1 transcript (1225 bp) in various tissues of human (a), rhesus monkey (b), crab-eating monkey (c), African-green monkey (d), and marmoset (e). *RPL32* (80 bp) indicates the positive control. M indicates the size marker. Primate cDNA samples are abbreviated as follows: CE: cerebellum; CO: colon; LI: liver; LU: lung; KI: kidney; PA: pancreas; ST: stomach. Structural analysis of transcript variants (f). Open and closed boxes represent the untranslated region of the exons and protein-coding region, respectively.

To investigate the expression pattern of isoform b transcript (containing the Plat_L3 element-derived exon), we performed comparative RT-PCR analysis in 6 human and marmoset tissues (cerebrum, colon, liver, lung, kidney, and stomach) and 7 rhesus monkey, crab-eating monkey, and African-green monkey tissues (cerebrum, colon, liver, lung, kidney, pancreas, and stomach). To amplify the isoform b-specific transcript in primate tissues, we designed primers specific to the 9th exon (sense primer) and Plat_L3-derived exon (antisense primer). We confirmed that these primer pair sequences were highly conserved in human and marmoset. The expected RT-PCR products (215 bp) were ubiquitously transcribed in all tested samples (Figures 3(a)–3(e)). Remarkably, an unexpected product (upper bands) was also ubiquitously expressed in rhesus monkey, crab-eating monkey, and African-green monkey. Although the unexpected bands were not detectable by gel electrophoresis of human and marmoset samples, we confirmed that the very weak bands were present in these species. Sequencing of the amplified product revealed that it was an intron-retained transcript variant (V1) (Figure 3(f)).

## 4. Discussion

### 4.1. TE-Exonization of LINE Elements during Primate Evolution

**Figure 4:**
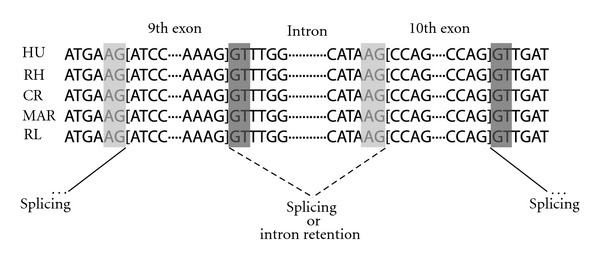
Boundary sequences of potential 3′ and 5′ alternative splice sites in various primates. Canonical splicing acceptor site “AG” and donor site “GT” are indicated in gray and dark gray, respectively. The sliced line indicates splicing, and the dotted line indicates splicing or retained intron.

A structural analysis revealed that Plat_L3 is integrated between the 9th and 10th exons in an intronic region of *ZRANB2*, where it provides an alternative splicing site that generates a new Plat_L3-derived exon via exonization (Figures 1 and 4). To determine when the Plat_L3 element was integrated, PCR amplification was performed in various primate genomes (Figure 2). Plat_L3 was integrated in all primate lineages, suggesting that Plat_L3 was integrated in a common ancestor prior to the divergence of simian and prosimian, possibly more than 63 million years ago. RT-PCR showed that the Plat_L3-encoded exon is transcribed in human, Old World monkey (rhesus monkey, crab-eating monkey, and African-green monkey), and New World monkey (marmoset) (Figures 3(a)–3(e)). Sequencing analysis showed that integrated Plat_L3 sequences are highly conserved in all primate lineages, in comparison to the adjacent intronic sequences and L3b element (Supplementary Figure  2). Moreover, canonical splicing sites (splicing acceptor and donor sites) are perfectly conserved from the hominoid to prosimian lineages (Figure 4). Therefore, perfectly conserved splicing sites of Plat_L3 and well-conserved Plat_L3 sequences could be exonized in *ZRANB2* gene transcripts in different primate lineages, including hominoids, Old World monkeys, and New World monkeys. We were unable to validate the Plat_L3-derived transcripts in prosimian samples, but, based on sequence analysis, we assume the existence of exonization events in ring-tailed lemur (Supplementary Figure  2).

### 4.2. Abundant IR Event in Old World Monkeys

An unexpected large band (1225 bp) detected by RT-PCR amplification was found in all tissues of Old World monkeys (rhesus monkey, crab-eating monkey, and African-green monkey) (Figure 3). Sequencing revealed this to be an alternatively spliced variant with the 9th intron retained and transcribed via an IR event [10]. Intriguingly, the intron-retained exon was detected at very low levels in human and marmoset monkey, unlike Old World monkeys. We suggest 2 alternative hypotheses of lineage-specific IR events and mixed mechanisms, including IR events and lineage-specific protection of nonsense-mediated mRNA decay (NMD).

In the previous model of IR events, high GC content in an intron sequence reduced the excision rate [24], and the retained introns are significantly shorter than nonretained introns [25]. However, we found that the 9th intron sequence of *ZRANB2* has a low GC content (approximately 30%) in human, rhesus monkey, and marmoset and was 1023 bp long, which is greater than the length of nonretained introns. Therefore, these older models could not explain the results of the IR event in ZRNAB2. The splicing acceptor and donor sites of the 9th and 10th exons were highly conserved in primates. Therefore, the IR event was not induced by weak signals of alternative splicing sites (Figure 4). Recent studies have shown that splicing is repressed by the binding of polypyrimidine tract-binding proteins (*PTB*) to specific sequence motifs (CUCUCU, UUCUCU, UUCCUU, and CUUCUUC), induced by IR events in *FOSB* [26]. We found that the *PTB*-binding consensus sequence UUCUCU 16 bp upstream from the 3′ end of the 9th intron was perfectly conserved from hominoid to prosimian (Supplementary Figure  2). Moreover, recent studies suggested regulation of *PTB* movement from the cytoplasm into the nucleus by phosphorylation [27]. These concepts are merged in the *ZRANB2* gene, where the IR event may occur via the *PTB*-binding sequence in human, rhesus monkey, crab-eating monkey, African-green monkey, and marmoset; however, the phosphorylation status of PTB may regulate the IR event in the primate lineage.

To explain the mixed mechanisms including IR events and lineage-specific protection of NMD, we first analyzed the relationship between IR events and NMD. In most cases, IR introduces premature termination codons (PTCs) into the mRNA, typically resulting in degradation by NMD [28]. The exon junction complex (EJC) NMD model is a well-known regulatory mechanism in mammals. The distance of PTC from the exon-exon junction is important; PTC located more than 50–55 nucleotides (nt) upstream of the last exon-exon junction causes mRNA decay by NMD, whereas PTC-located downstream of this boundary does not induce NMD [29]. The 9th exon of the V1 transcript containing the retained intron induced a PTC in the adjacent 5′ exonic region (Figure 3(f)). This PTC is located more than 50–55 nucleotides (nt) upstream of the last exon-exon junction. Theoretically, the V1 transcript should be degraded by the NMD mechanism in all tested primates; however, it was transcribed in all tissues of rhesus monkey, crab-eating monkey, and African-green monkey (Figures 3(a)–3(e)). Therefore, we suggest a lineage-specific protection event for NMD, specifically in Old World monkeys. Although a lineage-specific NMD protection mechanism has not been clearly established, a few studies have shown that the cytidine (C) to uridine (U) RNA-edited *APOB* mRNA was protected from NMD by the *APOBEC1*-*ACF*-editing complex [30]. The uORF-containing thrombopoietin (*TOP*) gene and nonsense mutation in the first exon of the *β*-globin (*HBB*) gene also escape NMD [31, 32]. Therefore, these elements specific to Old World monkeys may yield similar results. Supplementary Figure  2 illustrates the 7 Old World monkey-specific nucleotides. We believe that this sequence affects protection from NMD. However, further studies are needed to demonstrate the validity of this mechanism, including the relationship between specific nucleotides and NMD escape.

The species- and lineage-specific IR, NMD, and NMD escape mechanisms have not been demonstrated. A large number of IR studies have been performed in human and mouse. Therefore, experimental validation of our results in Old World monkeys (rhesus monkey, crab-eating monkey, and African-green monkey) suggesting that abundant IR events could be an attractive topic for IR and NMD research.

### 4.3. Three Isoforms of *ZRANB2*

Different transcripts are generated by the Plat_L3-derived exonization and intron retention events in *ZRANB2*. These transcripts encode Ctermini of varying size and amino-acid composition (Supplementary Figure  1). Isoform a (NM_203350.2), isoform b (NM_005455.4), and V1 encoded 330, 320, and 314 amino acids, respectively. The amino-acid sequences encoded by isoform b (SQVIGENTKQP) and V1 (FGFL) differ from the sequence in the C-terminus of isoform a. This region belongs to the SR domain, which is essential for nuclear localization of *ZRANB2* and is regulated by phosphorylation [33]. These functional features were demonstrated by an SR deletion study. However, more detailed deletion studies have not been performed. Although we did not perform a functional analysis, our results indicate that Plat_L3-exonization and intron retention events could yield various transcripts in the primate lineage. A previous study suggested that integrated full-length L1 elements in the intronic region of host genes could cause transcriptional interference (TI) by IR and exonization [34]. However, isoforms a and b and variant transcripts of the *ZRANB2* gene were not prematurely terminated by IR and exonization derived from the Plat_L3 element (Figure 3(f)). Our results also show that Plat_L3-exonized isoform b was transcribed in all tested human and monkey samples. The TI effect did not occur in *ZRANB2* by IR and exonization events derived from the Plat_L3 element. Therefore, we focused on the mechanism of gene diversity by IR and exonization.

## 5. Conclusion

We investigated and compared exonization derived from Plat_L3 element and IR events in the *ZRANB2* gene in human and monkeys (rhesus monkey, crab-eating monkey, African-green monkey, and marmoset). First, we confirmed that the Plat_L3 and L3b LINE elements in the intronic region of *ZRANB2* were integrated in human and all primate lineages (18 species, including hominoids, Old World monkeys, New World monkeys, and prosimian). RT-PCR experiments indicated that the Plat_L3-encoded exon was conserved in all tested tissues of human and the 4 monkeys; the IR event occurred only in Old World monkeys (rhesus monkey, crab-eating monkey, and African-green monkey). Transcript variants of *ZRANB2* genes derived from these events encoded different-sized products via 6-frame translation sequence analysis. Based on our results, we assume that IR and TE-derived exonization events are intriguing evolutionary factors that could enhance the transcriptome and protein diversity under limited genomic sources in the primate lineage.

## Supplementary Material

Figure S1: Amino acid analysis of *ZRANB2* transcript variants.
Figure S2: Multiple alignment analysis of Plat_L3 and L3b elements with various primate DNA sequences. Dark gray and yellow indicate Old World monkey-specific sequence and the PTB-binding motif, respectively.
