# Supplementary figures and images for: Intron Retention and TE Exonization Events in ZRANB2
Source: Comp Funct Genomics. 2012 Jun 17;2012:170208. doi: 10.1155/2012/170208 (PMC3384923; doi:10.1155/2012/170208)

FIGURE S1

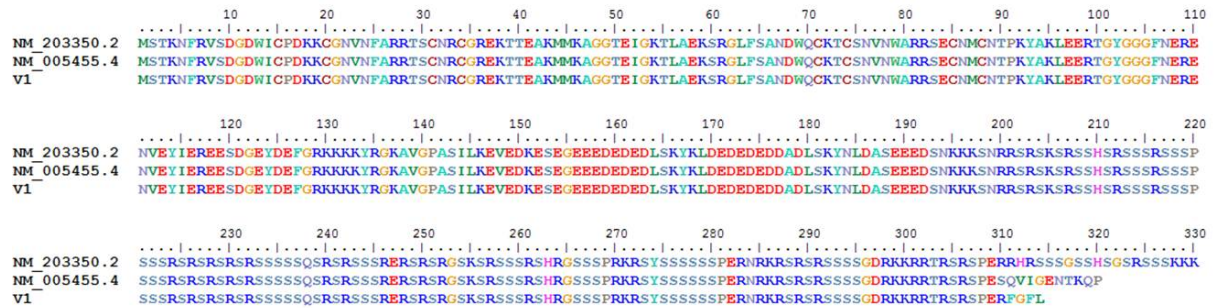

FIGURE S2

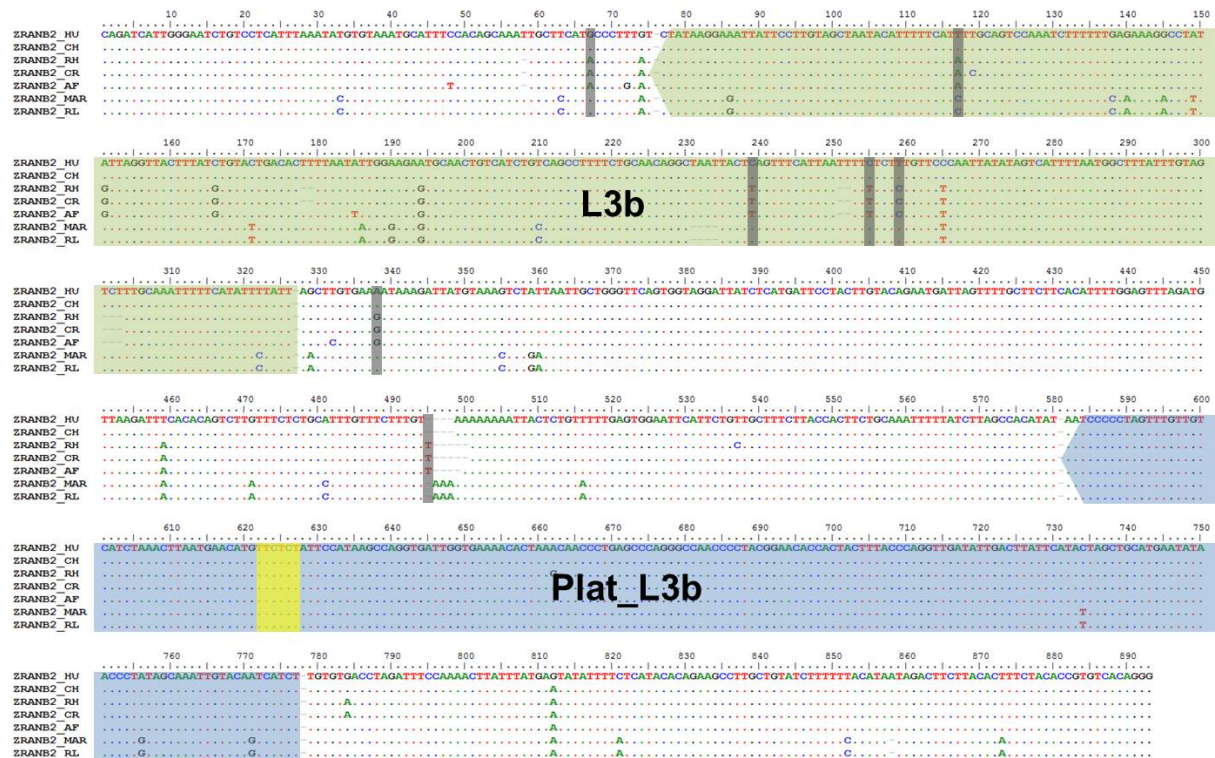

Supplement: Supplementary file 1 — Figure S1: Amino acid analysis of ZRANB2 transcript variants. Figure S2: Multiple alignment analysis of Plat_L3 and L3b elements with various primate DNA sequences. Dark gray and yellow indicate Old World monkey-specific sequence and the PTB-binding motif, respectively. [file 170208.f1.pdf]
